# Atrial remodelling and dysfunction in hypertrophic cardiomyopathy: prognostic role and therapeutic target

**DOI:** 10.3389/fcvm.2025.1620313

**Published:** 2025-07-08

**Authors:** Chiara Piazzai, Alessio Petrone, Andrea Stefanini, Flavio D’Ascenzi, Iacopo Olivotto, Matteo Cameli

**Affiliations:** ^1^ Unit Cardiomyopathies, Department of Clinical and Experimental Medicine, University of Florence, Florence, Italy; ^2^ Department of Medical Biotechnologies, Division of Cardiology, University of Siena, Siena, Italy; ^3^ Cardiology Unit, Meyer University Children Hospital, Florence, Italy

**Keywords:** hypertrophic cardiomyopathy, atrial myopathy, multimodal imaging, atrial fibrillation, septal reduction therapy, cardiac myosin inhibitors

## Abstract

**Introduction:**

Hypertrophic cardiomyopathy (HCM) is a common genetic cardiac disease marked by abnormal ventricular hypertrophy. Recent studies have highlighted that left atrial (LA) remodelling—including dilation, fibrosis, and functional impairment—plays a key role in disease progression and prognosis, notably increasing the risk of atrial fibrillation (AF) and stroke.

**Material and methods:**

This review article systematically examines published clinical, imaging, and interventional studies. The analysis focuses on identifying the determinants of atrial myopathy, its relationship with diastolic dysfunction and left ventricular outflow tract obstruction (LVOTO), and the effects of therapeutic interventions such as septal reduction therapy and cardiac myosin inhibitors.

**Results:**

The findings reveal that LA remodelling in HCM is characterized by increased LA volume, reduced atrial strain, and prolonged conduction times—all of which are strongly linked to the onset and recurrence of AF. Moreover, interventions that reduce LVOTO (e.g., surgical myectomy) have been shown to induce LA reverse remodelling and improve diastolic parameters. Emerging therapies, like cardiac myosin inhibitors, also improve LV function but present complex effects on atrial performance, with some evidence suggesting a reduction in atrial strain that warrants further investigation.

**Conclusion:**

Atrial remodelling is a significant marker of disease severity in HCM and an important independent predictor of adverse outcomes, including AF and cardioembolic events. Early detection through comprehensive multimodal imaging and timely therapeutic intervention can potentially mitigate these risks, making atrial myopathy both a critical prognostic factor and a promising therapeutic target.

## Graphical Abstract

**Figure:**
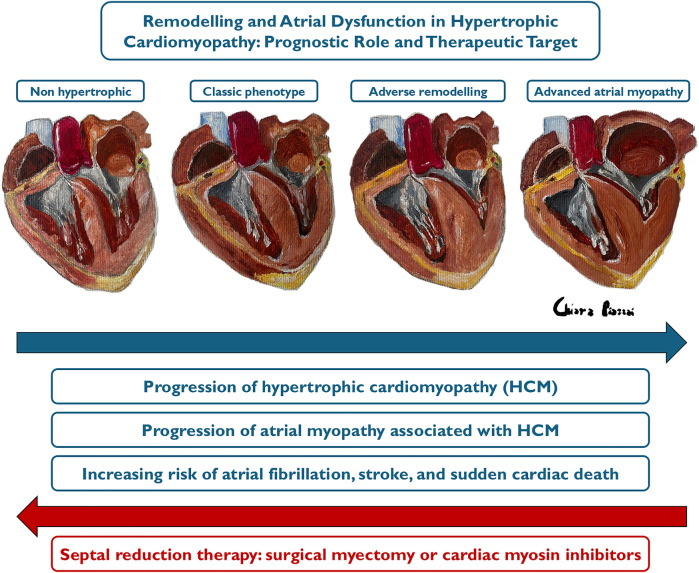

## Highlights

• Hypertrophic cardiomyopathy not only affects the left ventricle but also causes structural and functional changes in the left atrium, leading to atrial myopathy, a key marker of disease severity.
• Atrial myopathy significantly increases the risk of atrial fibrillation, which in turn raises the likelihood of stroke and worsens overall prognosis.
• Left atrium size and function are strong predictors of cardiovascular events, emphasizing the need for early intervention to reduce morbidity and mortality.
• ECG, echocardiography and cardiac magnetic resonance are crucial for early detection of atrial dysfunction.
• Surgical myectomy promotes reverse atrial remodelling by reducing left ventricular outflow tract obstruction and mitral regurgitation. Cardiac myosin inhibitors may offer similar benefits, but their effect on atrial remodelling is controversial due to potential negative inotropic effects on atrial function.

## Introduction

Hypertrophic cardiomyopathy (HCM) is the most common genetic cardiomyopathy, with a prevalence of approximately 1:500 in the general population (1). The disease exhibits autosomal dominant mendelian inheritance in about half of the cases, with variable penetrance; over 500 mutations have been described in 13 genes, primarily encoding for proteins of the cardiac sarcomere (2–4). HCM is characterized by abnormal hypertrophy of the left ventricular wall and is defined by the presence of unexplained left ventricular (LV) wall hypertrophy ≥15 mm, or ≥13 mm in the presence of family history or ECG abnormalities (4). Most HCM phenotypes present with asymmetric hypertrophy, primarily localized in the basal anterior septum, which can lead to systolic anterior motion (SAM) of the mitral valve leaflets, resulting in mitral regurgitation (MR) and dynamic LV outflow tract obstruction (LVOTO). Another hallmark feature of HCM is impaired diastolic function, characterized by reduced ventricular compliance and elevated LV filling pressures. These abnormalities lead to diminished exercise capacity and may ultimately progress to overt heart failure and increased risk of mortality. Indeed sudden cardiac death (SCD) is the most feared complication, particularly among individuals in the second and third decades of life, which is why several clinical predictors have been validated to identify high-risk patients who may benefit from primary prevention implantable cardioverter-defibrillator (ICD) therapy (5). Unfortunately, SCD may be the first manifestation of the disease in otherwise healthy and asymptomatic individuals, including athletes (6). While HCM is the most frequent cause of SCD in young individuals and can lead to functional disability due to heart failure (HF) and stroke, in most cases it does not cause significant symptoms or a major reduction in life expectancy. However, about 25% of cases may progress to “adverse remodelling” due to the progressive development of fibrosis, leading to thinning of the ventricular walls and a gradual reduction in the LV ejection fraction (EF). In a minority of patients, this can lead to relevant LV dysfunction with EF < 50% (7, 8). Although HCM has traditionally been considered a disease that primarily, if not exclusively, affects the LV, it is important to emphasize that it can also cause significant changes in the function and structure of the left atrium (LA), leading to a true “atrial myopathy”. This condition has been identified as a marker of disease severity and an important independent prognostic factor for cardiovascular events (9–11). Furthermore, it is the main contributor to the onset of clinically significant arrhythmias, such as atrial fibrillation (AF), which significantly increases the risk of stroke in HCM patients compared to the general population (12). Therefore, this review aims to examine the underlying mechanisms and consequences of atrial myopathy, exploring its prognostic value and proposing possible preventive strategies, with the goal of highlighting an aspect of HCM that is often overlooked.

## Prevalence and determinants of left atrial remodelling

**Figure 1:**
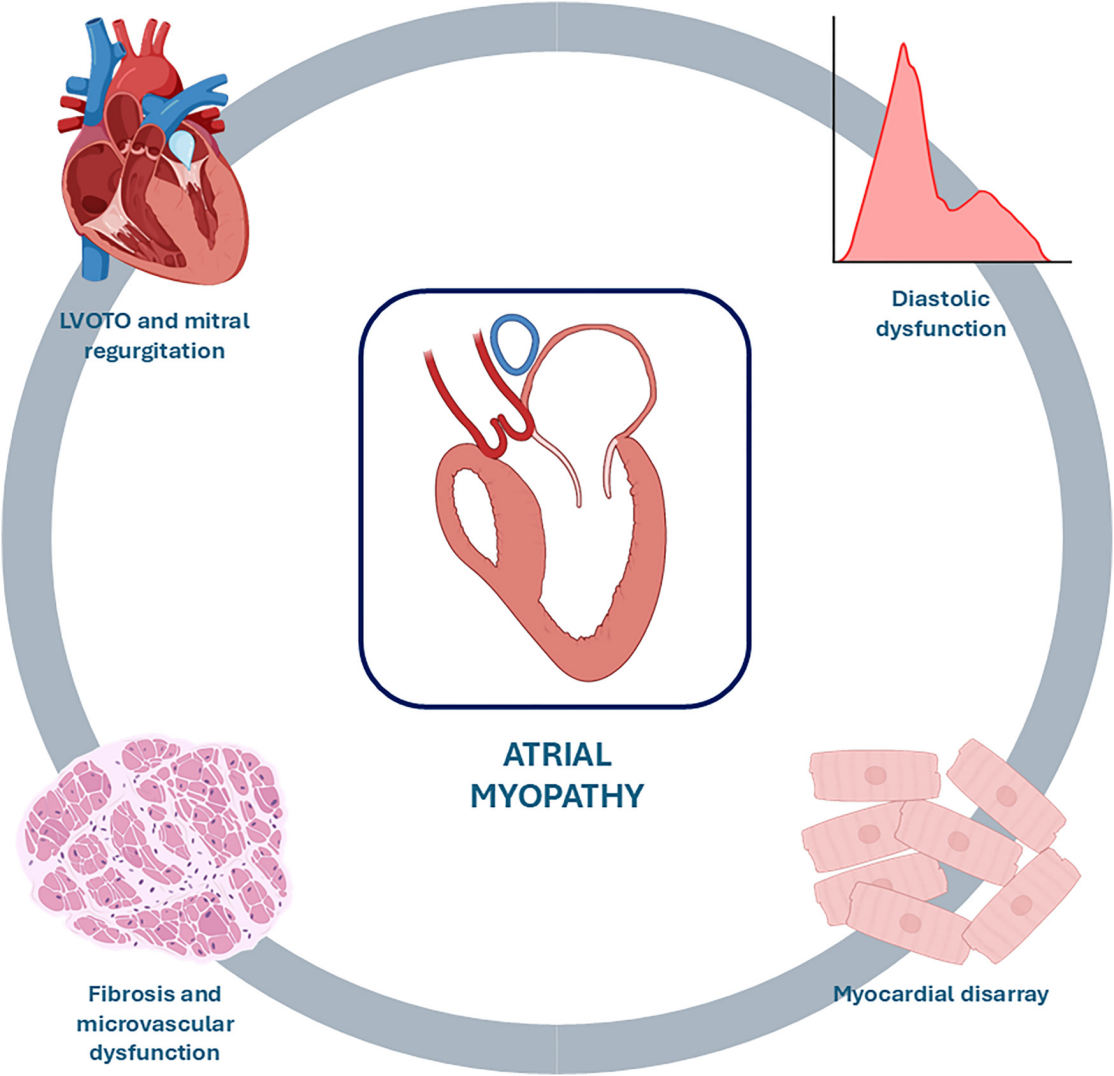
Causes of atrial myopathy. Prolonged exposure to mitral regurgitation, LVOTO, and myocardial disarray with resulting fibrosis, microvascular dysfunction, and diastolic dysfunction leads to adverse remodelling of the LA characterized by dilation and functional deterioration, a condition known as atrial myopathy. LVOTO, left ventricular outflow tract obstruction.

The size of the LA serves as a barometer for chronic left atrial pressure elevation, which results from the combination of diastolic dysfunction, MR, and atrial arrhythmias (13). In fact, prolonged exposure to MR, LVOTO, and LV remodelling, leading to high ventricular filling pressures and diastolic dysfunction, results in increased LA afterload, causing its adverse remodelling. This is characterized by atrial complex changes in contractility, electrophysiology and chamber architecture, which define the condition known as atrial myopathy (Figure 1), where ageing, oxidative stress and stretch, and inflammation lead to fibrosis, electrical remodelling and pro-thrombotic state, determining a close interplay among AF and stroke (Graphical Abstract) (14, 15).

### Mitral regurgitation and LVOTO

The primary phenotypic expression of obstructive HCM manifests through the characteristic elongation of the mitral valve leaflets, LV hypertrophy, and systolic hyper dynamism. These factors contribute to the phenomenon known as SAM of the mitral valve leaflets, in which one or both leaflets come into contact with the interventricular septum during ventricular systole, causing obstruction of normal blood flow in the left ventricular outflow tract (LVOT) towards the aorta (16). Additionally, the increased intraventricular pressure due to LVOTO can cause functional mitral insufficiency, with a regurgitant jet typically affecting the lateral and posterior wall of the LA. The severity of the regurgitation and the degree of the outflow gradient are dynamic and can be influenced by conditions that alter inotropism (e.g., physical exercise or the use of positive inotropic drugs), modify preload (e.g., dehydration, Valsalva manoeuvre, or change from squatting to standing position), or reduce afterload (e.g., physical exercise, vasodilator drugs, or septic states) (17). Symptoms associated with obstruction and mitral regurgitation include dyspnoea, chest pain, reduced exercise tolerance, presyncope, and syncope. LVOTO is not only an important pathophysiological component of HCM and one of the main determinants of atrial myopathy, but it is also associated with a worse prognosis compared to non-obstructive forms of HCM. In this context, a study involving 1,101 patients with HCM demonstrated that patients with LVOTO have a significantly higher risk of cardiovascular death, cardioembolic stroke, and progression to NYHA class III or IV (18).

### Diastolic dysfunction

A large proportion of patients with HCM experience HF symptoms despite normal systolic function and the absence of LVOTO and mitral insufficiency, identifying a phenotype of HF with preserved EF (HFpEF). This underscores the importance of myocardial relaxation and LV filling in generating symptoms in this disease (19). Indeed, in patients with HCM, diastolic dysfunction is an important component of the disease pathophysiology (20). The grading of diastolic dysfunction is crucial for the evaluation of patients with HCM and HFpEF, since the nature and extent of the disturbed physiology are able to explain patient's symptoms; hence, this grading should be assessed through the LV filling velocities (mitral E/A ratio) and the level of LV filling pressures [according to E/e' ratio, peak tricuspid regurgitation (TR) velocity, indexed left atrial volume (LAVi) and peak atrial longitudinal strain (PALS)] (21).

The origin of diastolic dysfunction lies primarily in cellular alterations, such as myocyte disarray, which are also associated with a state of hypercontractility and an inability to achieve complete relaxation. These microstructural alterations underlie myocardial hypertrophy, microvascular dysfunction, and fibrosis, factors known to increase myocardial wall stress in HCM patients. This causes an increase in LV filling pressures and LA afterload, resulting in elevated pressure in the LA to compensate for the reduced compliance of the LV (22). This process gradually leads to left atrial myopathy.

## Correlation with atrial fibrillation and cardioembolic stroke

AF is the most common sustained supraventricular arrhythmia in patients with HCM, with a clinical incidence of 2%–4% per year and a prevalence ranging from 19% to 30% in various cohorts, far exceeding rates in the general population of similar age (23). The prevalence of AF in HCM increases with disease progression, from subclinical phenotypes to end-stage disease, with a cumulative incidence over 40% in patients with LV dysfunction (24, 25). Moreover, up to 25% of patients with HCM, who are cardiac implantable electronic devices (CIEDs) carriers, experience paroxysmal and clinically silent episodes of AF (26, 27).

Due to the rigidity and reduced compliance of the LV, the contribution of atrial systole during diastole is typically increased in HCM patients compared to healthy individuals, both in obstructive and non-obstructive forms (28). Therefore, impairment of atrial function and the onset of AF leads to a reduction or loss of atrial systole, significantly influencing the risk of HF (25, 29). Patients with HCM and AF experience HF symptoms approximately 5 times more frequently than the general population, leading to increased rates of hospitalization, cardiovascular mortality, and a general deterioration in quality of life (25, 30, 31).

Thanks to the greater awareness of this condition, however, HCM is often diagnosed at an earlier stage, often before AF develops (32). Therefore, it is essential to identify patients with HCM who are at high risk for developing AF. LA size is a strong predictor for the development of AF, but even so, patients with HCM can develop AF in the absence of LA dilation, and conversely, not all patients with LA dilation will develop AF (12). Therefore, given the clinical importance and relevance of early AF diagnosis in HCM patients, several clinical risk stratification algorithms for developing AF have been proposed. Recently, a predictive model for the development of AF over the next 5 years (Hypertrophic Cardiomyopathy Atrial Fibrillation, HCM-AF Score, Table 1) has been validated in a large cohort of HCM patients, categorizing patients into three risk groups (low, intermediate, and high) (33). This score incorporates 4 variables (LA diameter, age, age at HCM diagnosis and presence of HF symptoms) and has been proven to perform better than other existing non-HCM specific tools (CHARGE-AF, HARMS_2_-AF, C_2_HEST, CHA_2_DS_2_-VA) for prediction of AF in patients with HCM. The authors of this study recommend increased attention to patients at high risk for AF, justifying a multi-level strategy that involves careful rhythm monitoring through periodic 48-hour Holter ECG or implantable loop recorder (ILR) implantation, and patient education regarding signs and symptoms of AF. In the largest external validation study to date, retrospectively conducted on 602 HCM patients without prior AF and followed for a median of approximately three years, the HCM-AF score demonstrated strong discriminatory power, maintaining consistent predictive accuracy across diverse subgroups, including White and non-White patients, obese and non-obese individuals, and gene-positive vs. gene-elusive patients. These findings reinforce the use of the HCM-AF score for personalized AF risk stratification in HCM. Accordingly, the 2024 AHA/ACC guidelines recommend validated tools such as the HCM-AF score for individualized AF risk assessment (34). Looking ahead, advances in artificial intelligence may drive a paradigm shift in risk prediction, as emerging machine learning models incorporating numerous variables show encouraging potential for even more precise assessment (35, 36).

**Table 1:** HCM-AF score for assessing the risk of atrial fibrillation in hypertrophic cardiomyopathy patients (33).

Clinical variable	Value	Score
LA anteroposterior diameter (mm)	24–29	+8
	30–35	+10
	36–41	+12
	42–47	+14
	48–53	+16
	54–59	+18
	60–65	+20
Age at the time of the visit (years)	0–9	+0
	10–19	+3
	20–29	+6
	30–39	+9
	40–49	+12
	50–59	+15
	60–69	+18
	70–79	+21
Age at the diagnosis of HCM (years)	0–9	+0
	10–19	−2
	20–29	−4
	30–39	−6
	40–49	−8
	50–59	−10
	60–69	−12
	70–79	−14
HF symptoms	Yes	+3
	No	+0

HCM-AF Score ≤17: low risk (<5% at 5 years).

HCM-AF Score 18–21: intermediate risk (5%–10% at 5 years).

HCM-AF Score ≥22: high risk (>10% at 5 years).

LA, left atrium; HCM, hypertrophic cardiomyopathy; AF, atrial fibrillation; HF, heart failure.

### Anticoagulation therapy

A large number of studies have shown that AF is associated with a dramatically higher risk of stroke in patients with HCM compared to healthy individuals (25, 30, 37). For this reason, according to the most recent European Society of Cardiology guidelines on Cardiomyopathies, anticoagulation therapy should be initiated in HCM patients diagnosed with AF, regardless of the CHA_2_DS_2_-VASc score (Class I, Level of Evidence B) (4).

However, it should be considered that the changes described in atrial structure and function lead to reduced intra-atrial flow, resulting in blood stasis, thereby triggering the Virchow triad, even in the presence of stable sinus rhythm (38). Indeed, recent studies have suggested that the incidence of ischemic cardioembolic stroke in patients with HCM and LA dilation may be similar between patients in sinus rhythm and those with AF, raising the question of whether patients with extreme remodelling and dysfunction of the LA should receive prophylactic anticoagulation even when in sinus rhythm (39). This phenomenon has been observed in various clinical scenarios and is a feature shared by other cardiomyopathies with hypertrophic characteristics, such as Fabry disease and cardiac amyloidosis, where atrial thrombi are widely documented even in patients maintaining sinus rhythm (40–42). From a therapeutic standpoint, although both vitamin K antagonists and direct oral anticoagulants are equally effective for thromboprophylaxis, the latter maintain a better safety profile and improved tolerability, making them the preferred option, unless contraindicated (43, 44). The role of left atrial appendage closure in HCM remains unknown.

### Antiarrhythmic therapy

Due to the clinical and hemodynamic impact of AF onset in patients with HCM, maintaining sinus rhythm is an important therapeutic goal. However, current antiarrhythmic therapy has significant limitations in terms of efficacy and safety, as adequate rhythm control can only be achieved in a minority of treated individuals (42% at 3 years of follow-up) (45).

Amiodarone is generally safe in the HCM population and may reduce the arrhythmic burden (45, 46). QT interval prolongation is rare, and proarrhythmic consequences are highly limited (45). However, long-term treatment can lead to hepatic, thyroid, and pulmonary toxicity, resulting in worse prognosis and treatment discontinuation (45). These issues are particularly important in this clinical context because most patients with HCM have a long-life expectancy.

Disopyramide is an older class IA antiarrhythmic drug commonly used to reduce LVOTO in HCM patients (46). It may represent a reasonable choice for rhythm control, although its overall efficacy is modest (35% after 3 years), due to its negative inotropic effect that facilitates the reduction of LVOT gradient (45). However, a significant proportion of patients on this treatment require discontinuation due to its anticholinergic effects.

Class IC antiarrhythmic drugs (such as flecainide and propafenone) are widely used in the general population for the prevention of AF recurrences. A small observational study of 48 patients with obstructive HCM showed a good safety profile in these patients treated with flecainide, with moderate improvement in obstruction and symptomatic status (47). The authors did not report any data on the efficacy of suppressing atrial or ventricular arrhythmias, and the small sample size did not allow for definitive conclusions. Therefore, their use is not yet indicated in HCM patients.

Catheter ablation is a more effective rhythm control strategy than antiarrhythmic therapy in the general population (48, 49). However, in patients with HCM, the efficacy of ablation is significantly reduced, with an increased incidence of AF recurrences (50–53). In this context, a recent study demonstrated that HCM is associated with a three-fold increase in the risk of AF recurrence following catheter ablation [hazard ratio (HR) 3.07, *p* < 0.001], compared to individuals without any form of cardiomyopathy (54). This is due to the persistence of mitral regurgitation, LVOTO, diastolic dysfunction, and resultant atrial myopathy, even after the procedure. Indeed, the success rate of a single catheter ablation is approximately 40% at 3–5 years, and around 50% after multiple procedures (55). It should be noted that the success rate of ablation varies depending on the timing: catheter ablation is more effective in younger, less symptomatic individuals with less atrial remodelling, and those with paroxysmal rather than persistent AF (55, 56). Procedurally, standard pulmonary vein isolation (PVI) is performed only in patients with paroxysmal AF, while in cases of persistent AF or extensive pathological atrial substrate, non-pulmonary triggers such as the posterior wall, roof, and mitral isthmus are typically targeted (57). One study reported similar efficacy between cryoablation and radiofrequency techniques, while data on emerging electroporation ablation are still lacking (58).

Electroanatomic mapping can be an excellent indicator of atrial health as it allows the assessment of any fibrotic areas, defined as low-voltage regions. In particular, a study by Professor Efremidis' group highlighted that patients with HCM and AF have significantly larger fibrotic areas compared to HCM patients in sinus rhythm and healthy individuals with AF. Additionally, the study found that an area of low voltage (≤0.25 mV) occupying more than 14.1% of the total LA surface was the best and only predictor of AF recurrence after catheter ablation, with excellent sensitivity and specificity (100%) (59).

Surgical ablation is a viable option in patients with HCM undergoing septal myectomy (60). Surgeons can perform classical PVI along with additional lesions aimed at the left atrial appendage, mitral isthmus, and roof and posterior wall segments. Surgical ablation is undoubtedly the most effective technique in HCM patients, as it has been shown that 75%-80% of patients are free from AF recurrence at follow-up (60). However, this outcome may be attributed not only to the direct effect of surgical ablation but also to the concomitant reduction in LVOTO and/or correction of MR, factors that significantly influence the atrial substrate.

## Evidence from multimodal imaging and prognostic significance

**Figure 2:**
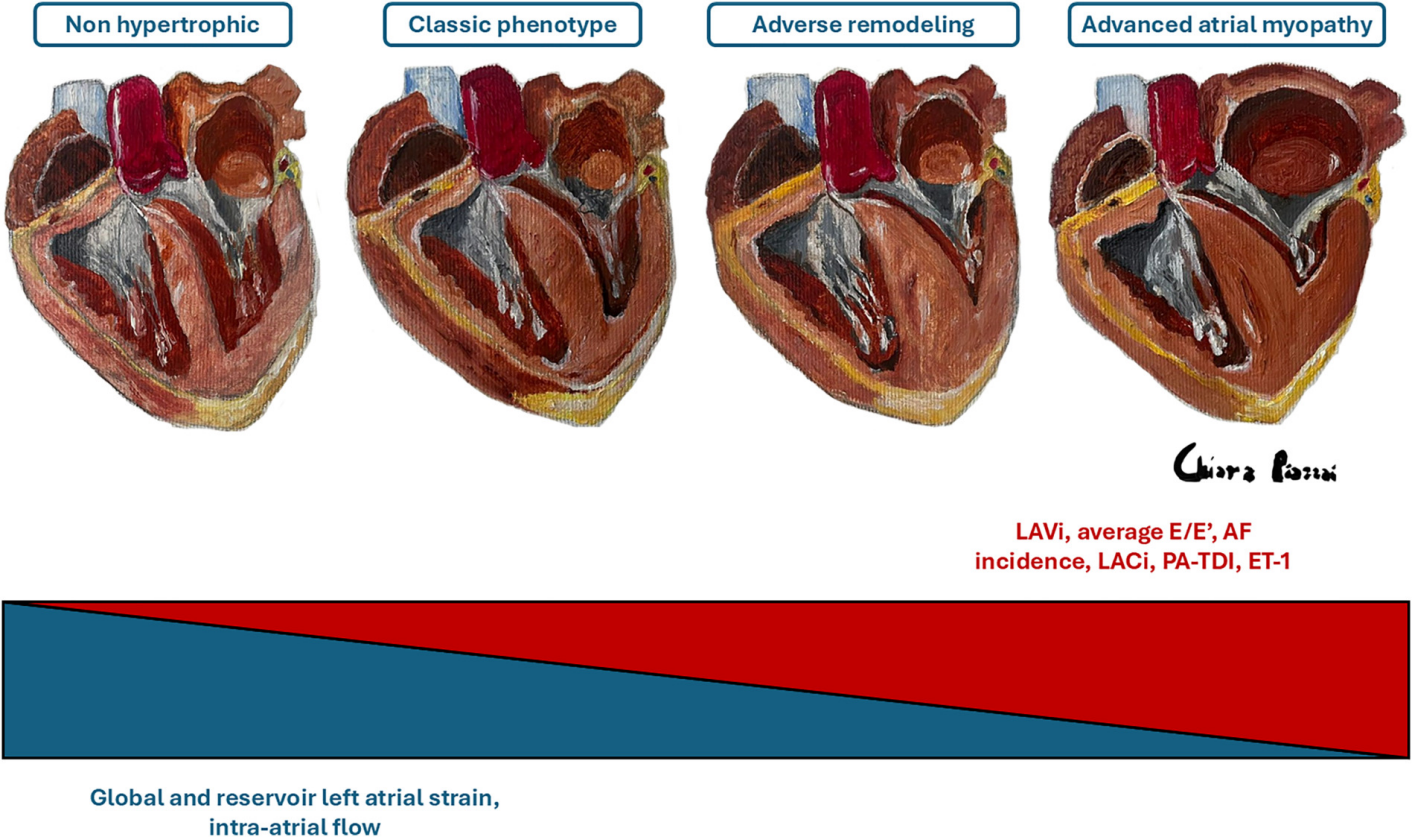
Evolution of atrial myopathy. The figure schematizes the main parameters that increase (in red) or decrease (in blue) as the severity of atrial myopathy progresses. ET-1, endothelin-1; AF, atrial fibrillation; LACi, left atrioventricular coupling index; PA-TDI, total atrial conduction time; LAVi, indexed left atrial volume. *Dr. Chiara Piazzai, Four Hearts on Canvas, Oil and Turpentine, 2024.*

**Figure 3:**
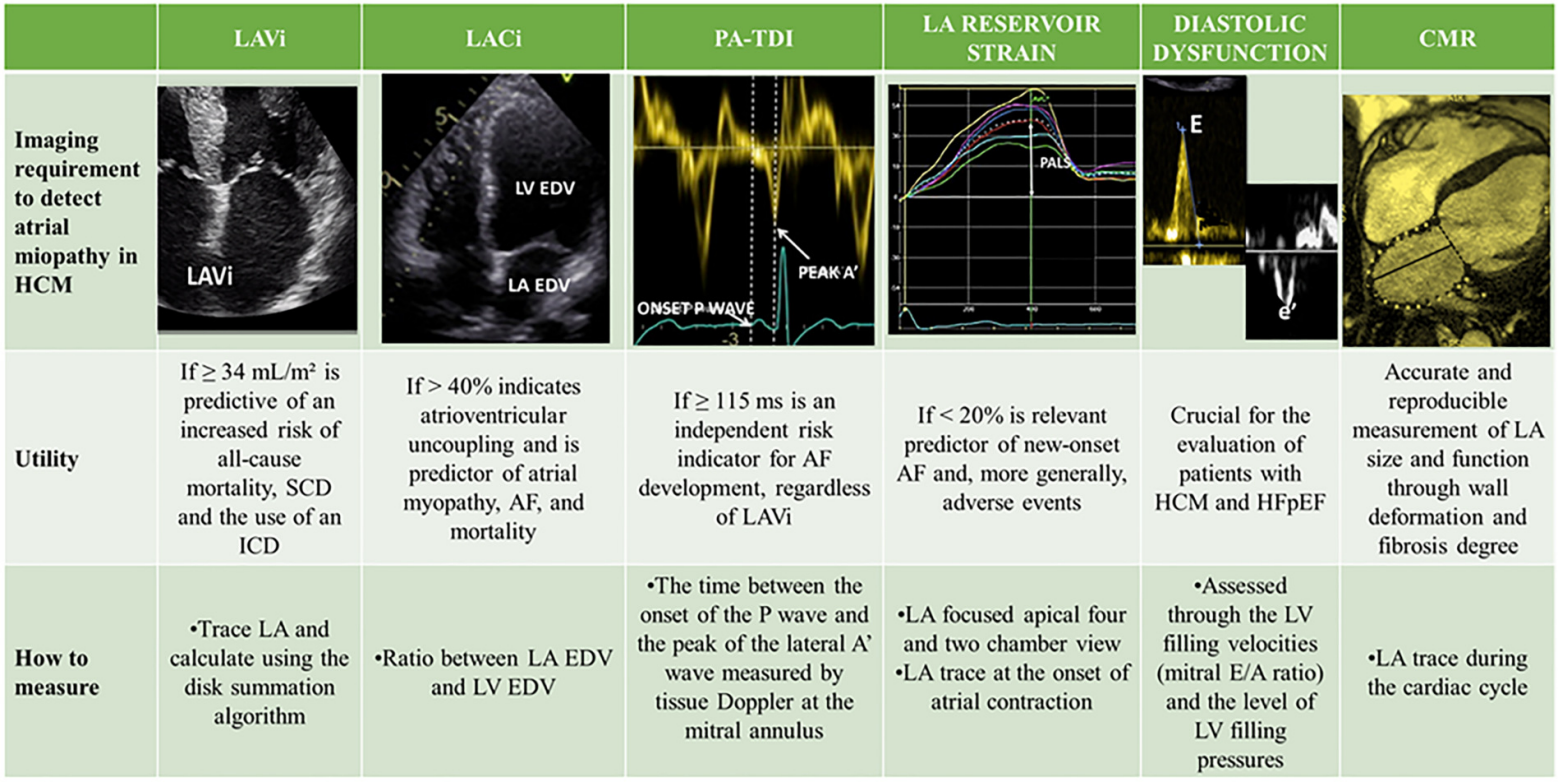
Multimodal imaging of atrial myopathy. AF, atrial fibrillation; CMR, cardiac magnetic resonance; EDV, end-diastolic volume; HCM, hypertrophic cardiomyopathy; HFpEF, heart failure with preserved ejection fraction; LA, left atrium; LACi, left atrioventricular coupling index; LAVi, indexed left atrial volume; LV, left ventricle; PA-TDI, total atrial conduction time; PALS, peak atrial longitudinal strain; SCD, sudden cardiac death.

The size of the LA is an independent risk factor for adverse outcomes in HCM patients, and an increased LA volume is associated with a higher risk of cardiovascular mortality, AF, thromboembolic stroke, and hospitalizations (10, 12, 31, 61, 62). In a review of 427 patients with HCM, Hiemstra et al. found that a LAVi ≥34 ml/m^2^ was predictive of an increased risk of all-cause mortality, heart transplantation, SCD and the use of an ICD (63). Specifically, in this review, LA dilation was shown to be an independent and strong predictor of SCD. For this reason, the LA anteroposterior diameter is an important parameter in the validated HCM-SCD Risk Score, the main risk score that guides the indication for ICD implantation in primary prevention in HCM patients (5). Therefore, timely detection of LA remodelling is a primary goal in the management of HCM to reduce mortality, prevent the onset of AF and cardioembolic stroke, and improve symptoms of HF (64). However, LA dilation in HCM represents an indicator of overt atrial myopathy (Figure 2), emphasizing the need for the development of new parameters capable of predicting the increase in LA size and its worsening in function (Figure 3) (65). Insights into this issue can be drawn from studies on LA function in athletes, which reveal that atrial size alone is insufficient to provide mechanistic information about atrial health. Notably, an increase in atrial size does not inherently indicate atrial dysfunction (66). Indeed, in athletes, despite significant atrial enlargement, the atria typically maintain normal reservoir function, myocardial stiffness, and dynamic adaptability to varying loading conditions. Consequently, the assessment of atrial function, rather than size alone, is crucial for accurately evaluating atrial myopathy (66).

### Role of ECG

Due to its low cost and ease of use, significant attention has been given in the literature to the identification of electrocardiographic parameters capable of predicting the onset of atrial remodelling and AF in patients with HCM. Among these parameters, P wave duration has emerged as the most reliable. In a study conducted by our group on 110 HCM patients in sinus rhythm, it was shown that a P wave duration greater than 140 ms was associated with an increased risk of AF, although with suboptimal sensitivity (56%) and specificity (83%) (67).

### Role of echocardiography

The LA and LV are directly coupled during ventricular diastole, and in the absence of mitral valve stenosis, their functions and filling pressures are linked. An increase in LA volume relative to LV volume at the end of diastole directly reflects impaired LV compliance. Following this concept, one study demonstrated that values of the left atrioventricular coupling index (LACi, defined as the ratio of LA to LV end-diastolic volumes) greater than 40% are indicative of atrioventricular uncoupling and are better predictors of atrial myopathy, AF, and mortality than simple LA size assessment (68). This suggests that an initial change in LV compliance, geometry, and pressures, leading to a reduction in LV volumes due to worsening hypertrophy and disease progression, could signal functional deterioration of the LA before its overt enlargement.

Significant mitral regurgitation has been shown to be associated with LA myopathy independently of the degree of LV diastolic dysfunction in HCM. Therefore, identification of moderate or greater mitral insufficiency, regardless of LA size, warrants greater attention and further investigation (69).

Moreover, in a study involving a cohort of 208 patients, total atrial conduction time (PA-TDI), a parameter evaluating the relationship between structural and electrical remodelling of the atrial myocardium, was significantly correlated with new-onset AF in HCM patients. PA-TDI is calculated as the time between the onset of the P wave on the ECG and the peak of the lateral A' wave measured by tissue Doppler at the mitral annulus. A PA-TDI value ≥115 ms was found to be an independent risk indicator for AF development, regardless of LAVi (70).

Finally, speckle-tracking echocardiography (STE) has emerged as a valuable tool for the early detection of LA dysfunction, even when LA dimensions are preserved. By measuring myocardial deformation throughout the cardiac cycle, LA strain offers a comprehensive assessment of atrial function. It is typically divided into three components (a) LA reservoir strain, reflecting LA expansion during ventricular systole and isovolumetric relaxation; (b) LA conduit strain, representing passive emptying of the LA during early diastole after mitral valve opening; (c) LA booster strain, indicating active LA contraction in late diastole (71). A decline in global LA strain and PALS, expression of maximal deformation at the end of the reservoir phase, often precedes the LA enlargement. Notably, reduced LA strain may be one of the earliest detectable abnormalities in carriers of HCM-related gene mutations, even before phenotypic expression of the disease (72). LAS not only correlates well with volumetric analysis of LA but also has the ability to detect atrial fibrosis and the development of AF as well as prevalence of MACE in patients diagnosed with HCM (73). Interestingly, a reduced global PALS was found to be the strongest predictor of invasively assessed severe LA fibrosis at cardiac biopsy in patients with end-stage HF, explaining the high burden of AF in these patients (74). Moreover, LA reservoir strain has been identified as a strong predictor of both new-onset AF and its recurrence following catheter ablation. Lack of significant improvement in LA strain after the procedure is associated with a higher likelihood of arrhythmia recurrence (75, 76). Finally, recent evidences suggest that a reduction in global LA strain <23.4% or in reservoir strain <20% are relevant predictors of new-onset AF and, more generally, adverse events, independent of LA size (73, 77, 78). As suggested by a recent meta-analysis, integrating LA strain into current risk prediction models could significantly improve individualized care and guide medical or procedural strategies in patients with HCM (73). Beyond the atrium, STE is also instrumental in evaluating LV mechanics, providing early markers of systolic and diastolic dysfunction. In a study of 56 patients with non-obstructive HCM, reduced longitudinal, circumferential, and radial LV strain values were detected before a decline in LVEF, highlighting the sensitivity of STE for subclinical myocardial dysfunction (79). Furthermore, the STE of the LV provides valuable insights regarding the AF risk in HCM patients. Indeed, in a prospective cohort of 250 HCM patients without prior AF, a global longitudinal LV strain value >–14% was a significant predictor of new-onset AF, underlining the role of LV mechanics in arrhythmia risk stratification. In conclusion, In conclusion, STE is a powerful, non-invasive imaging modality that allows simultaneous assessment of LA and LV function. It provides sensitive, reproducible, and clinically meaningful data that enhance both diagnostic evaluation and risk stratification.

### Role of cardiac magnetic resonance

Cardiac magnetic resonance (CMR) is an excellent tool as it allows highly accurate and reproducible measurement of LA size and function by assessing wall deformation and fibrosis degree, which is also fundamental to predict response to ablation therapy in AF patients (80–84).

Additionally, a novel CMR technique, 4D flow, has proven more effective than transoesophageal echocardiography in detecting blood stasis within the LA, a critical parameter for thrombus formation (85). However, there are currently no studies demonstrating an increased thromboembolic risk in HCM patients with reduced CMR intra-atrial flow. Although several CMR studies have been conducted in HCM patients, none have yet identified earlier and more reliable predictors of atrial myopathy and AF than LA enlargement or dysfunction. Future studies should also aim to compare reproducibility of LA strain measurements using CMR based techniques to standardize normal and abnormal values for LA strain in the HCM population.

### Role of genetics

In addition to morphological parameters, non-classical factors, such as genetic variants causing HCM, may play a crucial role in increasing the incidence of atrial myopathy and AF. Sarcomeric mutations have been shown to be associated with a higher prevalence and earlier onset of AF when compared to non-familial forms of HCM (86–88). Supporting this finding, it has been demonstrated that HCM patients with sarcomeric mutations, specifically in MYH7 and MYBPC3, exhibit a higher prevalence of low-voltage areas in the LA on electroanatomical mapping, indicative of LA scarring that may contribute to the AF burden, compared to genotype-negative HCM patients (6.1% vs. 2.3%; *p* < 0.001) (89). A study involving over 1,000 HCM patients, followed for an average of about 7 years, revealed that the incidence of AF in patients with sarcomeric mutations was approximately 19%, regardless of echocardiographic or clinical parameters. Notably, the presence of likely pathogenic or pathogenic mutations in the MYH7 gene had the highest incidence of AF after adjusting for age, sex, proband status, left atrial size, maximal wall thickness, and peak pressure gradient (HR 1.7, *p* = 0.009) compared to other HCM-related genotypes (90). A previously published analysis reported a peak AF prevalence of 47% in a family of 15 individuals with HCM caused by the Arg663His missense variant in the MYH7 gene (91). This represents a significantly higher incidence than in any other previously described HCM cohort (67).

However, numerous studies suggest that among sarcomeric mutations, those affecting genes encoding thin filament proteins are associated with a higher likelihood of advanced diastolic dysfunction and HF symptoms compared to those affecting thick filament proteins. In fact, a triphasic diastolic filling pattern of the LV is particularly common in HCM patients with this subclass of sarcomeric mutations, reflecting deep diastolic dysfunction that may predispose to LA remodelling and AF development (92). In this context, the missense variant Met228Thr in the ACTN2 gene, which encodes the α-actinin-2 protein, has been associated with familial mid-apical HCM and early-onset AF and atrioventricular blocks (93).

Finally, although less frequent and less widely studied, mutations in non-sarcomeric genes may increase the risk of AF and could therefore be considered potential modifiers of the HCM phenotype. In this regard, Orenes-Piñero et al. identified the 344T>C polymorphism in the CYP11B2 gene as an independent predictor of AF development in HCM patients (94). This polymorphism is associated with increased activation of the renin-angiotensin-aldosterone system, resulting in elevated serum aldosterone levels that may promote fibrosis and atrial remodelling.

Table 2 provides a comprehensive overview of all known genetic predispositions to early-onset AF reported in the literature to date.

**Table 2:** Genetic predisposition to atrial fibrillation (AF).

Gene	Mutation	Frequency/HR	Characteristics
Sarcomeric genes			
MYH7 (90)	Pathogenic or likely pathogenic	HR 1.72; *p* = 0.009, vs. MYBPC3 mutations	Obstructive HCM and early-onset AF
MYH7 (91)	c.12601G>A (p.Arg663His) missense mutation (autosomal dominant inheritance)	47% (7/15 members of the family) vs. 13% (*p* = 0.006) reported in HCM population with unknown genotypes and a similar age distribution	Obstructive HCM and early-onset AF
MYH7 or MYBPC3 (89)	Pathogenic or likely pathogenic	6.1% low-voltage areas in the LA at electroanatomical mapping (vs. 2.3% in genotype-negative HCM; *p* < 0.001)	Obstructive HCM
MYH6 (88)	Pathogenic or likely pathogenic	/	Early-onset AF and lower risk of mortality because it is predominantly expressed in the atria with a more isolated atrial involvement compared with those with an MYH7 variant
TTN (117, 118)	Pathogenic or likely pathogenic, truncating variant	HR 1.77; *p* < 0.001, vs. healthy people	Early-onset AF
MYL4 (119)	c.234delC frameshift deletion (autosomal recessive inheritance)	100% (8/8 members of the family)	Early-onset AF
TNNT2 (120, 121)	c.835C > T (p.Gln279Ter) nonsense mutation (autosomal dominant inheritance)	12% (1/8 members of the family)	Early-onset AF
ACTN2 (93)	c.683T > C (p.Met228Thr) missense mutation	36% (4/11 members of the family) with early-onset AF	Mid-apical HCM, early-onset AF, and AV block
Other genes			
CYP11B2 (94)	c.344T > C polymorphism	HR 3.31; *p* = 0.008, vs. healthy people	Early-onset AF due to increased activation of the renin-angiotensin-aldosterone system
JPH2 (122)	c.505G > A (p.Glu169Lys) missense mutation	100% (2/2 members of the family) with early-onset AF	Early-onset AF. Loss-of-function mutations in junctophilin-2 (JPH2) impair its ability to stabilize the sarcoplasmic reticulum, promoting calcium (Ca^2+^) leak and increasing susceptibility to atrial arrhythmias

AF, atrial fibrillation; HCM, hypertrophic cardiomyopathy.

### Circulating molecules

A study conducted on 375 HCM patients found that endothelin-1 (ET-1), a circulating protein, is an independent predictor of AF in this population. Specifically, ET-1 levels ≥0.285 pmol/L showed good specificity (75%) and sensitivity (56%) in predicting AF, even in the absence of LA dilation (95).

Finally, inflammation seems to play an important role in the pro-thrombotic state associated with AF (96). However, only a few studies have evaluated the importance of inflammatory biomarkers in directly predicting major clinical outcomes such as stroke and mortality. Therefore, further studies with larger sample sizes and important clinical endpoints are needed to assess the potential clinical significance of inflammatory biomarkers in AF.

## Impact of treatment

In light of the discussion above, it is crucial not only to identify atrial remodelling in its early stages to prevent severe complications such as AF, but it is equally important to understand how to halt and, if possible, reverse this process. As previously emphasized, LA remodelling in patients with HCM is mainly correlated with LVOTO, the severity of MR and diastolic dysfunction. Based on this evidence, it is intriguing to speculate that the removal of a triggering factor, such as LVOTO, could lead to subsequent reverse remodelling of the LA, with normalization of both dimensional and functional values. The main therapeutic options currently available are:

• Septal reduction therapy, which can be performed through either a surgical procedure (Morrow myectomy) or an interventional procedure (septal alcohol ablation);
• More recently, the use of cardiac myosin inhibitors.

These treatments aim to reduce LVOTO in order to significantly improve symptoms and potentially survival. However, at present, these therapies are often considered in symptomatic patients with obstructive HCM who are refractory to first-line medications (4). A list of studies related to this is available in Table 3.

**Table 3:** Main studies on the impact of LVOTO reduction on atrial myopathy.

Study	Procedure	Patients number	Mean age (years)	Follow up duration	LA diameter (mm)	LAVi (ml/m^2^)	E wave (cm/s)	E/A	E/e’	LA strain (%)
Septal reduction therapy										
Monteiro et al., 2007 (97)	Myectomy	150	44.5	3 years	From 43.6 to 45.2 (*P* = 0.06)	/	From 95 to 87 (*P* = 0.008)	From 1.53 to 1.44 (*P* = 0.30)	/	/
Menon et al., 2008 (98)	Myectomy	32	11.6	/	/	From 52.1 to 33.2 (*P* < 0.0001)	From 130 to 100 (*P* = 0.01)	From 1.9 to 1.3 (*P* < 0.0001)	Mean, From 20.7 to 10.8 (*P* < 0.001)	/
Moravsky et al., 2013 (99)	Myectomy	66	54	6–18 months	/	From 48 to 37 (*P* < 0.05)	From 80 to 78 (*P* > 0.05)	/	Lateral, from 11 to 8 (*P* < 0.05)	/
Finocchiaro et al., 2016 (101)	Myectomy (31) and alcohol septal ablation (9)	40	50	1 years	/	From 63 to 55 (*P* < 0.001)	From 91 to 92 (*P* = 0.92)	/	Mean, from 15 to 16 (*P* = 0.48)	/
Cavigli et al., 2018 (123)	Myectomy (71) and alcohol septal ablation (55)	126	53	5 years	From 50 to 47 (*P* = 0.027)	/	/	/	/	/
Nguyen et al., 2018 (100)	Myectomy	656	56	2 years	/	From 47 to 38 (*P* < 0.001)	/	From 1.15 to 0.95 (*P* = 0.002)	Mean, from 11.9 to 9.1 (*P* < 0.001)	/
Ha et al., 2023 (113)	Myectomy	44	52.8	1 year	/	From 61.5 to 49.1 (*P* < 0.001)	From 90 to 80 (*P* = 0.011)	/	Septal, from 25.7 to 23.0 (*P* = 0.049)	Global, from 24.4 to 30.5 (*P* = 0.004)
Cardiac myosin inhibitors										
Ho et al., 2020 (106)	Mavacamten in non-obstructive HCM	40	54	24 weeks	/	From 37.3 to 38.7 (*P* = 0.90)	/	/	Mean, from 14.1 to 11.5 (*P* = 0.50)	/
Hegde et al., 2021 (114)	Mavacamten	123	58.5	30 weeks	/	From 40.0 to 32.5 (*P* < 0.0001)	From 88 to 81.6 (*P* = 0.06)	/	Mean, from 17.5 to 13.9 (*P* < 0.0001)	/
Saberi et al., 2021 (111)	Mavacamten vs. placebo	35	60	30 weeks	/	Reduction of 10.3 (*P* < 0.001)	/	/	/	/
Cremer et al., 2022 (112)	Mavacamten	56	59.8	16 weeks	/	From 41.3 to 36.1 (*P* = 0.005)	/	From 1.4 to 1.3 (*P* = 0.57)	Mean, from 17.1 to 13.7 (*P* < 0.001)	/
Tian et al., 2023 (108)	Mavacamten	54	52.4	30 weeks	/	From 43.3 to 29.5 (*P* < 0.001)	/	/	/	/
Wessly et al., 2023 (115)	Mavacamten	15	70	30 days	/	From 39.5 to 35 (*P* = 0.37)	From 91 to 84 cm/s (*P* = 0.12)	From 1.00 to 1.10 (*P* = 0.15)	Mean, from 18 to 15 (*P* = 0.005)	Reservoir, from 27.5 to 22 (*P* = 0.012)
Desai et al., 2023 (107)	Mavacamten	56	59.8	56 weeks	/	From 41.3 to 35.8 (P not specified)	/	/	Septal, from 19.6 to 15.5 (P not specified	/

HCM, hypertrophic cardiomyopathy.

### Septal reduction therapy

A study involving 150 HCM patients undergoing surgical myectomy found a significant improvement in LV filling parameters, with a reduction in the mitral E-wave velocity (from 95 to 87 cm/s, *p* = 0.008) (97). Menon et al. retrospectively analysed data from a paediatric sample (ages 1–22 years, *n* = 32) who underwent septal myectomy for obstructive HCM. In this study, LAVi decreased from 52.1 ± 2.2 to 33.2 ± 11.9 ml/m^2^ (*p* < 0.001), the E/A ratio decreased from 1.9 ± 0.6 to 1.3 ± 0.5 (*p* < 0.001), and the tissue Doppler velocity at the medial mitral annulus (E′ medial) increased from 6.2 ± 1.9 to 13 ± 2.6 cm/s (*p* = 0.043) (98). Moravsky et al., in a study involving 66 patients with obstructive HCM, demonstrated significant reverse remodelling of the LA (LAVi decreased from 48 ± 16 to 37 ± 13 ml/m^2^, *p* < 0.05) after myectomy (echocardiographic follow-up of 6–18 months), with symptom relief and a reduction in obstruction (99). These results were confirmed by a large study of 653 patients who underwent surgical myectomy with a 24-month follow-up. This study showed a reduction in LAVi from 48 to 43 ml/m^2^ after the first year, and to 38 ml/m^2^ at the end of follow-up (*p* < 0.001), highlighting that LA reverse remodelling is an ongoing process years after the procedure (100). This large study also noted a significant improvement in diastolic function, with a decrease in the mitral E/A ratio from 1.15 to 0.95 (*p* = 0.002) and a decrease in mean E/e' ratio from 11.9 to 9.1 (*p* < 0.001) (100). Finally, it is important to mention a study by Finocchiaro et al., as it analysed a sample of around 40 patients who underwent both surgical myectomy and alcohol septal ablation. Consistent with previous trials, this study demonstrated that septal reduction therapy was associated with significant reverse remodelling of the LA and a major reduction in associated symptoms (101). However, it observed a trend towards greater reduction in LA volume after myectomy compared to alcohol septal ablation. This highlights how myectomy, often associated with mitral valve repair (another important determinant of atrial myopathy) is more effective in reducing filling pressures compared to alcohol ablation (102). In contrast to earlier studies, this one did not observe a significant improvement in diastolic function, particularly in tissue Doppler parameters such as E/e′. This may be due to the different demographic characteristics of the cohort, as the population studied had a longer disease history with more advanced atrial myopathy, associated with more significant LVOTO and MR, where diastolic dysfunction may have become fixed and no longer influenced by factors such as LVOTO.

These studies indicate that LA reverse remodelling is directly related to the reduction of the LVOT gradient and the severity of MR in patients with obstructive HCM. Since LA size reflects LV filling pressures, these results suggest that removal of the obstruction reduces atrial pressures, leading to actual reverse remodelling, particularly in the earlier stages of the disease. Therefore, it is crucial to also assess latent obstruction using stress echocardiography: the detection of positive hemodynamic criteria (exercise-induced hypotension and exercise-induced LVOTO >50 mmHg at peak exercise stress) allows the prediction of future development of progressive HF symptoms and the detection of the disease at an earlier stage, before obstruction becomes manifest (103, 104).

### Cardiac myosin inhibitors

In recent years, the pathophysiology of HCM has been progressively clarified, leading to a paradigm shift in treatment. This has marked a transition from an era of symptomatic therapies to one of targeted therapies that can actually modify the natural course of the disease. For HCM, these therapies include cardiac myosin inhibitors such as Mavacamten and Aficamten, small molecules that act directly at the sarcomeric level on the primary pathophysiological alteration underlying the disease, improving cardiomyocyte relaxation. The development of this type of therapy represents a very promising option for patients with HCM, as it has shown substantial hemodynamic, clinical, electrical, and structural improvement in both obstructive and non-obstructive HCM (105–110). These data offer extremely promising prospects regarding the long-term effects of these molecules. Therefore, numerous studies are ongoing to evaluate the impact of these molecules on atrial remodelling. The results obtained so far indicate that treatment with Mavacamten, similar to surgical myectomy, not only improves LVOT gradients but also results in an improvement in diastolic function parameters (111–113). In particular, the largest study to date, involving 251 patients with symptomatic obstructive HCM, showed that patients treated with Mavacamten had a significant reduction in LAVi, from 40 to 32.5 ml/m^2^ (*p* < 0.0001), a decrease in the lateral E/e' ratio from 15.0 to 11.2 (*p* < 0.0001), and a reduction in the degree of MR, thanks to a higher incidence of complete resolution of the mitral SAM (47% increase compared to the placebo group, *p* < 0.0001) (114).

Although studies demonstrate a positive effect of cardiac myosin inhibitors on atrial remodelling, the incidence of AF in this patient group does not seem to improve as markedly. Some small cohort studies on Mavacamten-treated patients indicate that the typical negative inotropic effect of the drug also occurs at the atrial level, resulting in a reduction of LA strain and of LA reservoir strain, both validated parameters and predictors of AF risk (115, 116). Therefore, further research on larger populations is needed to evaluate the long-term impact of myosin inhibitors on AF incidence.

In conclusion, treatment with cardiac myosin inhibitors induces LA reverse remodelling, leading to improvements in diastolic function and MR, similar to the effects observed with surgical myectomy. However, unlike myectomy, myosin inhibitors have a negative impact on atrial inotropism, as demonstrated by the results of preliminary studies on small cohorts, which show a significant reduction in LA strain. It is still unclear whether the positive effect of reverse atrial remodelling or the negative effect of atrial inotropism predominates in determining the incidence of AF. Further studies based on real-world clinical experience could clarify this issue.

## Limitations

While this review highlights the potential role of atrial myopathy in the pathophysiology and clinical course of HCM, it is important to acknowledge several limitations.

First, current evidence does not definitively establish atrial myopathy as a critical prognostic determinant of HCM severity. Although an association between atrial structural and electrical remodelling and adverse outcomes, particularly AF, is consistently observed, causality remains unclear. Furthermore, it is uncertain whether targeting atrial myopathy directly could meaningfully alter the natural history of HCM. Most available studies are observational, and interventional data addressing whether treatment of atrial myopathy improves long-term outcomes in HCM are lacking.

In addition, much of the literature focuses on surrogate markers, such as atrial size or fibrosis on imaging, which may not fully capture the complexity of atrial remodelling. There is also considerable heterogeneity in the definitions and methods used to assess atrial myopathy, making comparisons across studies difficult.

Finally, this review may be limited by publication bias and the predominance of data from tertiary care centres, potentially reducing generalizability.

Future studies are needed to clarify the prognostic significance of atrial myopathy in HCM and to determine whether it represents a meaningful therapeutic target beyond rhythm control in patients with AF.

## Conclusions

In patients with HCM, factors such as SAM of the mitral valve, LVOTO, increased LV filling pressures, and genetic predisposition contribute to LA dilation and fibrosis, leading to the development of a true atrial myopathy. This condition has been associated with an increased risk of cardiovascular events, all-cause mortality, and the incidence of AF. Therefore, it is crucial to identify strong predictors of atrial myopathy through the use of multimodal imaging, including ECG, echocardiography and CMR, to detect the early onset of the disease. In this context, LA strain has proven to be a particularly promising non-invasive tool, more sensitive than traditional volumetric analysis, as a significant reduction in LA reservoir strain often precedes the evident enlargement of the LA. From a therapeutic perspective, septal reduction therapies, particularly surgical myectomy, have been shown to be highly effective in halting this progression and promoting LA reverse remodelling, in addition to improving diastolic function. Data regarding the use of myosin modulators are promising but still inconclusive and somewhat discordant.
